# The Role of the Rho/ROCK Pathway in Ang II and TGF-β1-Induced Atrial Remodeling

**DOI:** 10.1371/journal.pone.0161625

**Published:** 2016-09-09

**Authors:** Li-Juan Liu, Feng-Juan Yao, Gui-Hua Lu, Cheng-Gui Xu, Zhe Xu, Kai Tang, Yun-Jiu Cheng, Xiu-Ren Gao, Su-Hua Wu

**Affiliations:** Department of Cardiology and Department of Ultrasonography (Feng-Juan Yao), the First Affiliated Hospital, Sun Yat-Sen University, Guangzhou, China; University at Buffalo - The State University of New York, UNITED STATES

## Abstract

**Objectives:**

To study the role of the Rho/ROCK pathway in Ang II and TGF-β1-induced atrial remodeling.

**Methods and Results:**

A canine atrial fibrillation (AF) model was established by rapid atrial pacing (RAP) of the left atrium. The roles of TGF-β1, the RhoA/ROCK signaling pathway and connective tissue growth factor (CTGF) in atrial remodeling were studied via both in vitro and in vivo experiments. Each of the dogs that received RAP developed persistent AF within 4 weeks. The mRNA expression levels of TGF-β1 (1.32±0.38), Collagen-I(1.33±0.91), CTGF(5.83±3.71), RhoA(1.23±0.57) and ROCK-1 (1.02±0.27) in the left atrium were significantly increased following 4 weeks of RAP. Angiotensin II (Ang II) induced the proliferation of atrial fibroblasts and up-regulated the expression of both CTGF and ROCK-1 in a dose-dependent manner. Simvastatin and Y27632 reversed Ang II-induced CFs proliferation, as well as ROCK-1(0.89±0.05 and 1.27±0.03, respectively) and CTGF (0.87±0.04 and 0.91±0.02, respectively) expression. The expression mRNA of ROCK-1(1.74±0.13) and CTGF (2.28±0.11) can upregulated by TGF-β1, and down-regulated by Simvastatin (1.22±0.03 vs 2.27±0.11), Y27632 (1.01±0.04 vs 1.64±0.03), Los (1.04±0.11 vs 1.26±0.05), respectively. Losartan and Simvastatin attenuated the effects of TGF-β1, inhibited RhoA activity as opposed to RhoA protein expression. Y27632 had no effect on either the expression or the activity of RhoA.

**Conclusions:**

The increased expression of profibrotic factors (CTGF, ROCK1 and Smad2/3) played an important role in our RAP-induced AF model. Increased atrial profibrotic factors involve the activation of either the TGF-β1/RhoA/ROCK-1 or the TGF-β1/Smad2/3 signaling pathway.

## 1. Introduction

Atrial fibrillation (AF) is the most common cardiac arrhythmia encountered in clinical practice and is common among the elderly, with an annual incidence of approximately 3% among patients 85 years of age and older[1]. Underlying heart disease may result in substantial morbidity[2]. AF is often triggered by rapid electrical activity originating from small areas that are typically located between the left atrium and the pulmonary veins[3, 4]. New treatment approaches such as catheter ablation have been used to successfully treat AF. However, said therapies are effective in only approximately 60% of patients, and the risk of recurrence is 30–50% during the first year following ablation[5]. Interstitial fibrosis and fibrotic bi-atrial substrates have been observed in cardiac specimens of patients with AF, findings suggestive of the causal role of fibrosis with respect to AF[6]. Fibrosis initially serves as an important adaptive response; however, increased atrial fibrosis contributes to the development of conduction abnormalities, resulting in electrical vulnerability. Electrical remodeling subsequently contributes to cardiac structural alterations. The molecular mechanisms underlying AF-induced atrial fibrosis remain unclear. The interrelated pathways apparently include the rennin-angiotensin system, TGF-β1[7], ERK[8] and oxidative stress pathways[9].

TGF-β1 activity has been observed during pathologic cardiac remodeling in a variety of animal models. Furthermore, the overexpression of TGF-β1 preferentially results in the fibrosis of atrial cells[10]. TGF-β1 is a multifunctional growth factor that has profound regulatory effects on many developmental and physiological processes. Disruption of the mouse TGF-β1 gene results in multifocal inflammatory disease[11]. New therapeutic targets of atrial fibrosis had focus on downstream of the TGF-β1 signaling. CTGF is a downstream factor of TGF-β1[12, 13] in the setting of Ang II-induced cardiac fibrosis; the blockade of the Ang II receptor decreases fibrosis[12]. TGF-β1 participates in the initial, acute phase of inflammation and repair following MI, whereas CTGF is associated with the ongoing fibrosis of the heart[14]. Ang II stimulation upregulates both TGF-β1 and CTGF expression in atrial fibroblasts and myocytes[15]; inhibition via ARB blunts atrial fibrosis in animal models[16, 17] and decreases the incidence of AF in human patients[18]. The RhoA/ROCK-1 pathway, which is downstream of TGF-β1, is another key mediator in tissue fibrosis[19]. The downregulation of Rho GTPase activity is associated with the decreased expression of TGF-β1, connective tissue growth factor, and type III collagen[20]. In recent years, accumulating evidence has demonstrated that the RhoA/ROCK-1 signaling pathway is involved in the development of the chronic inflammatory fibrosis of cardiovascular disease.

The role of atrial remodeling in AF and the mechanism underlying remain unknown. It is unclear whether the Rho pathway is involved in atrial fibrosis, downstream of the RAS. The relationship between the TGF-β1/RhoA/ROCK-1 pathway and the TGF-β1/Smad2/3 pathway in atrial remodeling must also be elucidated. In the present study, we aimed to determine the regulatory factors involved in the development of AF, as well as to develop a means of either attenuating or preventing AF.

## 2. Methods

### 2.1 Materials

Angiotensin II, Simvastatin, and Pentobarbital sodium were each purchased from Sigma (Sigma, USA). Losartan and Y-27632 were obtained from Selleck (Selleck Chemicals, USA). TGF-B1 was purchased from PROSPEC (PROSPEC, Israel). The ROCK-1, CTGF, Collagen–I (α1), TGF-β1, and Smad 2/3 antibodies were obtained from Santa Cruz Biotechnology (Santa Cruz, Delaware Avenue, CA). The RhoA pull-down was provided by Cytoskeleton (Cytoskeleton, USA). All DNA probes were synthesized by Invitrogen (Invitrogen, USA). The HE and Masson trichrome stains were both purchased from Maxin (Fuzhou, Fujian, China). The hydroxyproline detection kits were purchased from the Nanjing Jiancheng Bioengineering Institute (Nanjing, Ghina). Fetal calf serum and Dulbecco's modified Eagle medium (DMEM) were obtained from Gibco BRL (Gaithersburg, MD). All buffer solutions used in this study were prepared in our lab. Additional commonly used chemicals and materials were purchased from standard commercial sources.

### 2.2 Animal treatment

All experimental protocols involving the use of animals conformed to the Guide for the Care and Use of Laboratory Animals and complied with the guidelines specified by the Chinese Heart Association policy pertaining to research animal use and the Public Health Service policy pertaining to the use of laboratory animals. The experimental protocols were reviewed and approved by our institutional ethics committee (Approval No: IACUC-2011-0704). The beagles were purchased from Kangda Animal Science and Technology (Guangdong, China). The animals were maintained in the Animal Experiment Center of Sun Yat-sen University in temperature-controlled animal housing with 12 h alternating light and dark cycles and were provided adequate nutrition and water ad libitum.

#### 2.2.1 Left atrial rapid pacing

Fifteen beagles of both sexes (12–16 kg) were randomized into a pacing group (n = 9) and a control group (n = 6). Left atrial rapid pacing was performed as described previously, with some modifications[17]. The beagles were anesthetized using pentobarbital sodium (30 mg/kg) and intubated and ventilated using a volume-cycled ventilator (PA-500, PUAO, Nanjing, China). The ventilation was set to 4.2 L/min; the tidal volume was 0.3 L/min, and the rate was 16 breaths/min. One point six million units of penicillin were administered intravenously via the great saphenous vein. Body temperature was maintained at 37.0°C. The heart rate was monitored throughout the operation. The left thoracic cavity was opened via the sternum between the second intercostal and fourth intercostal spaces; the heart was subsequently exposed using a dilator. An electrode catheter (Temporary cardiac pacing lead, No: 2594–43, Tyco International Ltd. USA) was sutured to the left atrium for both pacing and recording in both groups. The distal ends of the electrode leads were tunneled subcutaneously, exposed at the neck, and connected to a pacemaker (an output of 6 V with a 1.0 ms pulse duration, 400 beats/min, Guangzhou Academy of Sciences, China) in a jacket.

The pacing group was maintained on high rate left atrial pacing (400 beats/min) for 4 weeks, with a brief break to allow for the measurement of both electrophysiological and mechanical parameters. The control group underwent an identical surgical procedure but did not undergo RAP. Postoperative care included the administration of both antibiotics and analgesics.

#### 2.2.2 ECG recordings

Electrocardiograms were recorded on days 1, 7, 14, 21 and 28 following surgery. An electrophysiological study was performed at 4 weeks following surgery, as was the case in previous studies[17, 21].

#### 2.2.3 The preparation of the atrial samples

At the end of the experiment, all beagles were sacrificed with euthanized using pentobarbital sodium (30 mg/kg), and their hearts were removed and weighed. The atria were subsequently removed and cut into three (upper, meddle, lower) sections. Each section was divided equally into ten pieces. One piece was used for the hydroxyproline content analysis. Three pieces were paraffin-embedded for hematoxylin and eosin staining, Masson's trichrome staining and immunohistochemistry analysis. The remaining pieces were frozen in liquid nitrogen and maintained at -80°C for both mRNA and protein analysis. A hydroxyproline content analysis was performed using a hydroxyproline kit according to the manufacturer’s instructions.

### 2.3 Western blotting

Fifty micrograms of total protein from homogenized atrial tissue were solubilized for 5 min at 95°C in loading buffer (1% SDS, 30% glycerol, 0.8 mM dithiothreitol, 1 mM Tris-HCl, pH 6.8, and 2% bromophenol-blue). The samples were subsequently loaded onto a 10% SDS-PAGE gel for electrophoresis and transferred to PVDF membranes. The membranes were blocked using 5% nonfat dry milk in PBS (containing 0.05% Tween 20) and incubated overnight at 4°C with the primary antibodies (monoclonal mouse anti-ROCK-1, anti-CTGF, and anti-Collagen-I, Santa Cruz, Delaware Avenue, CA). Following washing, the membranes were incubated with either an anti-rabbit or an anti-mouse IgG, HRP-linked antibody (1:3000; DAKO Real EnVision, Dako, Copenhagen, Denmark) for 2 hours at room temperature. Detection was performed using an ECL Western blotting kit (Thermo Fisher Scientific, Waltham, MA, USA). The band densities were quantified using Image J software (National Institutes of Health, Bethesda, MD, USA).

### 2.4 Immunohistochemical staining

For the immunohistochemical analyses, the sections were deparaffinized and digested using 0.05% subtilisin. The inactivation of endogenous peroxidase activity was performed via incubation in 3% H_2_O_2_ in methanol for 30 minutes. Following several washes in phosphate buffered saline (PBS), the slides were heated in a microwave oven at 121°C for antigen retrieval. After being cooled at room temperature and washed with PBS, the sections were subsequently incubated with blocking solution for one hour at room temperature. Following PBS washing, the tissues were bordered using a pap-pen before being incubated with mouse monoclonal antibodies against Collagen I (α1), ROCK-1, CTGF, TGF-β1 and anti-SMAD 2/3 (Santa Cruz, Delaware Avenue, CA) for 30 minutes at room temperature in accordance with standard protocols. The histological sections stained with HE and Masson trichrome solution were analyzed via light microscopy. A minimum of 3 sections per atrial site were examined.

### 2.5 RNA isolation and amplification

Total RNA was extracted from the atrial tissue in accordance with standard techniques using an RNeasy Fibrous Tissue Mini Kit and Micro Kit (QIAGEN, Hilden, Germany). The isolated RNA was quantified via UV spectrophotometry (Agilent Technologies, Boeblingen, Germany). Quantitative real-time polymerase chain reaction (RT-qPCR) was used to validate the levels of desmoplastic factor expression. As much as 1 μg of RNA per sample was reverse transcribed into cDNA using oligo-dT_15_ primers and M-MLV reverse transcriptase (TaKaRa, Dalian, China) according to the manufacturer’s instructions. All primers were designed using Primer 5 software. The experiments were performed using Power SYBR Green PCR Master Mix (TaKaRa, Dalian, China). All measurements were performed in duplicate. The relative quantification of expression was performed using the “2^-ΔΔCT^ method”[22] via the utilization of glyceraldehyde-3-phosphate dehydrogenase (*Gapdh*) for normalization.

### 2.6 Cell culture

Atrial tissue freshly obtained from Neonatal Sprague Dawley (SD) rat hearts. Neonatal SD rat were killed by cervical dislocation and hearts excised and placed in ice-cold isolation solution for mincing and digesting via incubation with 0.25% trypsin digest solution (Sigma, USA) for 4 h at 37°C. The atrial cardiac fibroblasts cells were collected via centrifugation, washed with DMEM (supplied with 0.05% bovine serum albumin (BSA)) and resuspended in full growth medium (FGM, DMEM supplemented with 10% fetal calf serum (FCS), 2 mmol/L L-glutamine, 100 μg/mL penicillin G, 100 μg/mL streptomycin and 0.25 μg/mL amphotericin). The cells were plated into cell culture flasks for 30 min to allow the fibroblasts to adhere, followed by the removal of any non-adherent cells. The atrial CFs were cultured until they reached 80–90% confluence in fresh full growth medium, in a humidified atmosphere of 5% CO_2_ at 37°C, and subsequently passaged via trypsinization. The experiments were performed using early passage cells (passages 2–5).

#### 2.6.1 The effect of Ang II on atrial CF proliferation

The atrial CFs were seeded into 96-well plates (5×10^3^ cells per well) in 150 μL of full growth medium. Following overnight incubation, the cells were growth-arrested in serum-free medium (SFM) for 48 h. The SFM was subsequently replaced with 150 μL of minimal growth medium (medium containing 2.5% FCS) containing the appropriate drugs. Four time points (6 h, 12 h, 24 h, and 48 h following the addition of the drugs) were designated in this study. Following the culture, viable cells were detected using an MTT assay as described previously[23]. The experiments were performed on cells from different passages to rule out the effects of non-specific passage. Similar proliferative responses were observed using cells from different passages (data not shown).

The atrial CFs were divided into the four following treatment groups, and each group was divided into several subgroups: group 1 was used to investigate the effect of Ang II on atrial CF proliferation. Group 2 was treated with Losartan (10^−8^, 10^−7^, 10^−6^, 10^−5^, 10^−4^ mol/L), both with and without Ang II (10^-6^mol/L). Group 3 was treated with Simvastatin (0.01μM, 0.1 μM, 1μM, 10μM, 100μM), both with and without Ang II (10^-6^mol/L). Group 4 was treated with Y27632 (10^−8^, 10^−7^, 10^−6^, 10^−5^, 10^−4^ mol/L), both with and without Ang II (10^-6^mol/L).

#### 2.6.2 Ang II related signaling pathway studies

The atrial CFs were seeded into 12-well plates (5×10^4^ cells per well) in 1500 μL of full growth medium. When the cells reached 80–90% confluence, they were growth-arrested in serum-free medium for 24 h, followed by 24 h of drug treatment. The SFM was subsequently replaced with 1500 μL of full growth medium for another 24 h culture. Two time points (4 h and 6 h following the addition of the drugs) were designated in this study.

The cells were divided into the following 8 groups: group 1 (control, without treatment), group 2 (TGF-β1, 5 ng/mL, 4 h and 21 h), group 3 (Y27632, 10 μM, 21 h), group 4 (Y27632, 10 μM, 21 h, TGF-β1, 5 ng/mL, 4 h), group 5 (Sim, 0.1 μM and 1 μM, 21 h), group 6 (Sim, 1 μM, 21 h, TGF-β1, 5 ng/mL, 4 h), group 7 (Los, 10^−6^ mol/L, 21 h, TGF-β1, 5 ng/mL, 4 h), and group 8 (Los, 10^−6^ mol/L, 21 h, Sim, 1 μM, 21 h, TGF-β1, 5 ng/mL, 4 h). Following treatment, the cells were collected, and the mRNA expression levels of ROCK-1 and CTGF were measured via RT-PCR. The expression levels of CTGF and phosphorylated-Smad2/3 were analyzed via Western blotting. RhoA activity was detected using a RhoA kit (Cytoskeleton, CO, USA) according to the manufacturer’s instructions.

The effect of the RhoA/ROCK-1 signal pathway on the Ang II induced expression of CTGF was subsequently studied. The cells were treated as follows: group 1 (control, without treatment), group 2 (Ang II, 10^-6^mol/L, 10^-7^mol/L, 10^-8^mol/L, 4 h and 21 h), group 3 (Y27632, 10 μM, 21 h, Ang II, 10^-6^mol/L, 21 h), group 4 (Sim, 1 μM, 21 h, Ang II, 10^-6^mol/L, 21 h), group 5 (Los, 10^−6^ mol/L, 21 h, Ang II, 10^-6^mol/L, 21 h), and group 6 (Los, 10^−6^ mol/L, 21 h, Sim, 1 μM, 21 h, Ang II, 10^-6^mol/L, 21 h). Following treatment, the cells were collected, and the mRNA expression levels of both ROCK-1 and CTGF were analyzed via RT-PCR. The expression levels of CTGF and ROCK-1, TGF-β1 were analyzed via Western blotting.

### 2.7 Statistical analysis

The quantitative data were presented as the means±standard deviations (mean±SDs). Comparisons between the quantitative data were made using the *t*-test, whereas those for the qualitative data were tested using the X^2^ test. Differences in continuous variables were examined via one-way ANOVA. SPSS 13.0 was used for the statistical analysis. *P* < 0.05 was considered statistically significant.

## 3. Results

### 3.1 The successful induction of AF and the electrophysiological parameter changes

AF was induced by S1S2 (250 ms, 300 ms, and 350 ms) and burst (600 bpm) stimulation, which was repeated 3 times. Eleven cases of paroxysmal AF were observed 36 times following S1S2 stimulation in the pacing group, whereas only 1 case was observed in the control group. Fifteen events of paroxysmal AF and 12 cases of persistent AF were induced via burst stimulation in the pacing group. However, only 6 cases of paroxysmal AF and no cases persistent AF were induced via burst stimulation in the control group. The incidences of paroxysmal AF in the control group and the pacing group were 33.3% (6/18) and 55.6% (15/27), respectively. The Incidences of permanent AF in the control group and the pacing group were 0% and 44.4% (12/27), respectively. Each of the dogs that received electrical pacing developed persistent AF within 4 weeks.

There was no difference in either the P interval or the P-A interval (*p =* 0.71, data not shown). The ERPAs at different pacing cycle lengths (250 ms, 300 ms, and 350 ms) were 134.46±21.30 ms (P<0.05 vs Group C), 126.66±10.00 ms (P<0.01 vs Group C) and 132.44±21.54 ms (P<0.05 vs Group C), respectively. The ERPAV increased from 160.00±0.00 ms to 167.78±4.41 ms (P<0.01vs Group C). CSNRT increased from 103.33±2.58 ms to 115.44±8.24 ms (P<0.01 vs Group C). The AVN-Wen increased from 328.33±24.01 bpm to 294.44±26.03 bpm (P<0.05 vs Group C) (Table 1).

**Table 1:** The changes in ERPA, ERPAV, CSNRT and AVN-Wen (x±s).

PCL ms	Group C(n = 6)		Group P(n = 9)		*P*	*P’*
	0 week	4 week	0 week	4 week		
**ERPA(250 ms)**	153.33±4.22	155.00±10.48	153.33±18.03	134.46±21.30**#	0.002	0.049
**ERPA(300 ms)**	150.00±6.32	143.33±10.32	142.22±12.02	126.66±10.00**##	0.001	0.008
**ERPA(350 ms)**	158.33±7.53	153.33±8.16	152.22±21.67	132.44 ±21.54**#	0.000	0.043
**ERPAV(350ms)**	156.67±8.16	160.00±0.00	157.78±6.67	167.78±4.41**##	0.003	0.001
**CSNRT(350ms)**	100.83±5.85	103.33±2.58	96.66±8.29	115.44±8.24**##	0.004	0.004
**AVN-Wen(bpm)**	325.00±15.16	328.33±24.01	335.55±25.05	294.44±26.03**#	0.034	0.024

Note. PCL: Pacing cycle length; ERPAs: atrial effective refractory periods; ERPAVs: atrioventricular nodal effective refractory periods; CSNRTs: corrected sinus node recovery times; AVN-Wens: Wenckebach points.*P<0.05, **P<0.01vs Before Pacing after 4weeks, ^#^*P’*<0.05,^##^*P’*<0.01 vs Group C after 4weeks.

Significant structural changes were observed in the heart following electrical pacing. The tissue adjacent to the atrium exhibited significant dilatation. Following 4 weeks of pacing, the left atrial anteroposterior diameter (LAap), left atrial upper-bottom diameter (LAud) and left atrial left-right diameter (LAh) were 24.33±3.39 mm (P<0.05), 27.11±4.01 mm (P<0.05), and 28.44±4.69 mm (P<0.05), respectively. The right atrial upper-bottom diameter (RAud) was 22.33±2.60 mm (P>0.05) at week 4, and no changes in the ventricular dimensions were observed (Table 2).

**Table 2:** The changes in echocardiographic indices before and after rapid atrial pacing (mm, x±s).

	Group C(n = 6)		Group P(n = 9)		*P*	*P’*
	0 week	4 week	0 week	4 week		
**LAap**	19.67±2.06	19.83±1.72	20.33±1.50	24.33±3.39#*	0.010	0.042
**LAud**	21.50±2.07	23.50±1.05	23.22±3.67	27.11±4.01#*	0.029	0.047
**LAh**	22.00±1.55	24.17±2.64	23.22±3.11	28.44±4.69*	0.010	0.065
**RAh**	18.00±2.00	18.50±1.05	17.22±2.82	21.22±7.39	0.095	0.392
**RAud**	21.50 ±3.39	21.33±2.16	20.22±2.49	22.33±2.60	0.062	0.451

Note. LAap: left atrial anteroposterior diameter; Laud: left atrial upper-bottom diameter; LAh: left atrial left-right diameter; Rah: right atrial left-right diameter; RAud: right atrial upper-bottom diameter.*P<0.05, **P<0.01vs Before Pacing after 4 weeks, ^#^*P’*<0.05, ^##^*P’*<0.01 vs Group C after 4

### 3.2 The elevated expression of hydroxyproline of the heart following RAP

**Fig 1:**
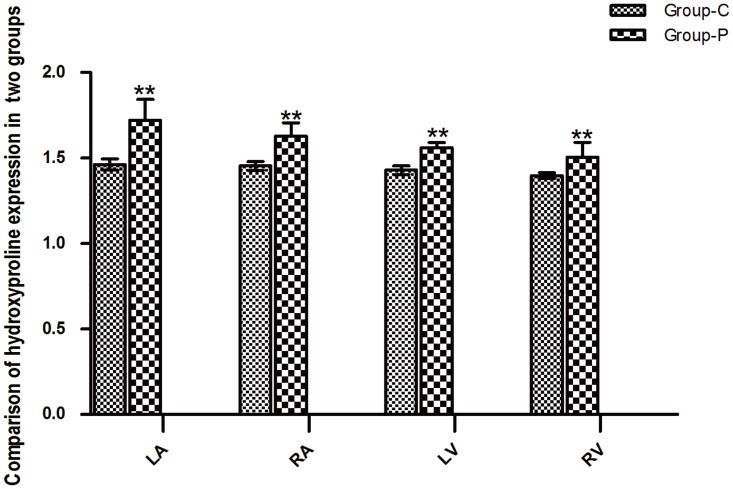
Comparison of hydroxyproline expression between the two groups. LA, left atrium; RA, right atrium; LV, left ventricle; RV, right ventricle. *P<0.05, **P<0.01. (n = 6 for Group C, n = 9 for Group P).

Following 4 weeks of RAP, the expression levels of hydroxyproline at different anatomic locations of the heart were analyzed. The results indicated that the expression of hydroxyproline was elevated following RAP (Fig 1). Compared with Group C, the expression of hydroxyproline was increased in the left atrium (1.72±0.12 vs 1.46±0.03, P<0.01), the right atrium (1.63±0.08 vs 1.45±0.03, P<0.01), the left ventricle (1.56±0.03 vs 1.43±0.03, P<0.01) and the right ventricle (1.51±0.09 vs 1.39±0.02, P<0.01) in group P; however, the expression levels of hydroxyproline of the left atrium was significantly increased compared with the other parameters (P<0.01). No difference in hydroxyproline expression was observed between the left and the right ventricle (P>0.05).

### 3.3 The effects of the elevated expression of the profibrotic factors on atrial remodeling

**Fig 2:**
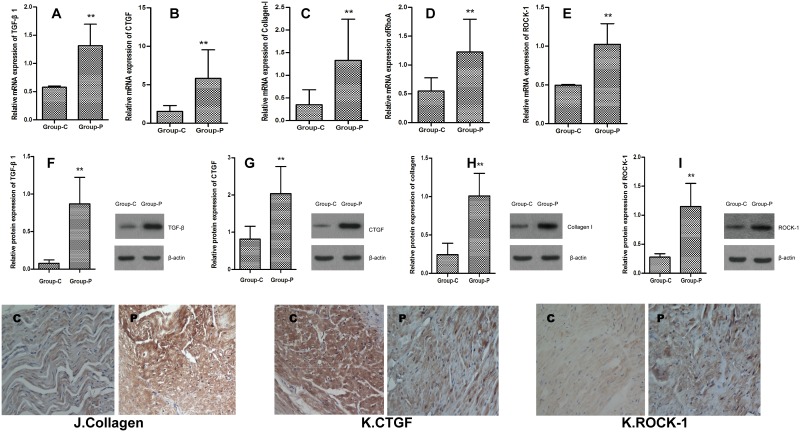
The upper panel depicts mRNA expression levels of TGF-β1 (A), CTGF (B), collagen-I (C), RhoA (D) and ROCK-1 (E) in the canine left atrium following 4 weeks of RAP. The middle panel depicts the protein expression levels of TGF-β1 (F), CTGF (G), collagen I (H) and ROCK-1 (I) in the canine left atrium following 4 weeks of RAP. The lower panel depicts the immunohistochemical results pertaining to collagen-I (J), CTGF (K) and ROCK-1 (L) in the hearts (×400). C, control group; P, pacing group. *P<0.05, **P<0.01 vs group C. (n = 6 for Group C, n = 9 for Group P).

The mRNA expression levels of TGF-β1 (1.32±0.38 vs 0.58±0.02), Collagen-I (1.33±0.91 vs 0.35±0.33) and CTGF(5.83±3.71 vs 1.53±0.76) in the left atrium were significantly increased following 4 weeks of RAP (Fig 2A–2C). The mRNA expression levels of RhoA (1.23±0.57 vs 0.55±0.23) and ROCK-1 (1.02±0.27 vs 0.49±0.01) were elevated in the pacing group (Fig 2D and 2E). The protein expression levels of TGF-β1(0.87±0.35 vs 0.08±0.05), CTGF(2.04±0.72,0.82±0.34), Collagen-I(1.01±0.29 vs 0.24±0.15) and ROCK-1(1.15±0.39 vs 0.28±0.06) were elevated following RAP (Fig 2F–2I). An immunohistochemical analysis demonstrated that the expression levels of Collagen-I, CTGF and ROCK-1 were increased in the left atrium in the pacing group (Fig 2J and 2K).

### 3.4 The pathological changes in the heart occurred following electrical pacing

**Fig 3:**
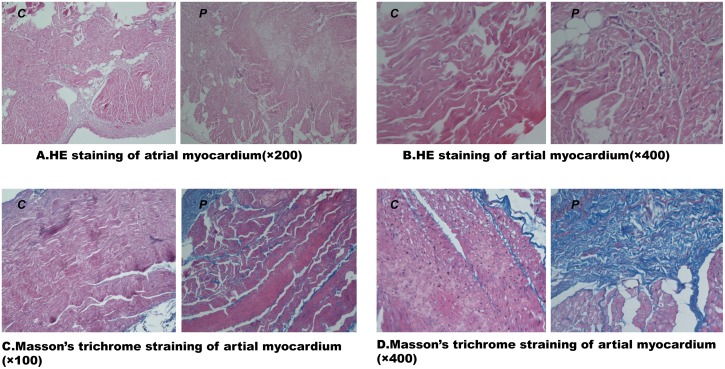
Representative HE staining (up panel) and Masson’s trichrome staining (down panel) of the atrial myocardium. (A) Structurally normal atrial myocardium in the control group and severe fibrosis in the pacing group (×100). (B) Atrial myocardium with HE staining under ×400 light microscopy. (C) A small amount of collagen is visible within the atrial myocardium of the control group, and an increased amount of collagen is visible within the atrial myocardium following pacing (×100). The red areas represent myocytes, and the blue areas represent collagen. (D) Atrial myocardium with Masson’s trichrome staining under ×400 light microscopy. C, control group; P, pacing group. (n = 6 for Group C, n = 9 for Group P).

The representative histological sections of the atrial myocardium of each group are depicted in Fig 3. Following 4 weeks of RAP, disordered and randomly distributed cells were observed throughout the section. The numbers of nuclei and fibroblasts were also increased following pacing. Masson staining for collagen demonstrated a significant increase in the level of fibrosis in the pacing group compared with the control group, as well as elevated collagen expression in the left atrium (Fig 3).

### 3.5 The RhoA/ROCK-1 pathway mediated Ang II induced cardiac fibroblast proliferation and CTGF expression in the atrial CFs

**Fig 4:**
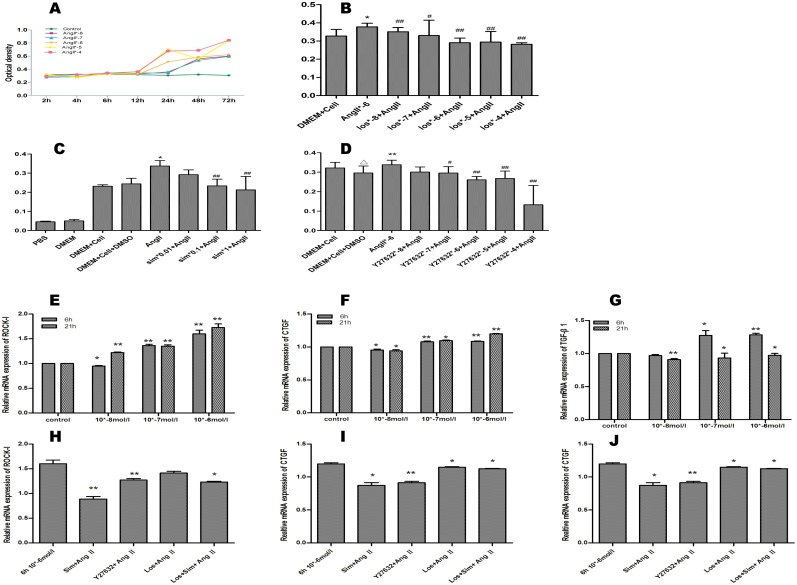
The effect of the Ang II pathway on atrial CF proliferation (upper panel) and the effect of Ang II on the mRNA expression of the AF factors in the atrial CFs (lower panel). (A) The effects of different concentrations of Ang II on the proliferation of the atrial CFs. (B, C, D) The effects of different concentration of Los (B), Sim (C) and Y27632 (D) on Ang II induced cell proliferation. Ang II upregulated the expression of ROCK-1 (E), CTGF (F) and TGF-β1 (G). Sim, Los and Y27632 downregulated the Ang II induced expression of TGF-β1 (J), CTGF (I) and ROCK-1 (H). *P<0.05, **P<0.01 vs Group C, #*P*<0.05, ##*P*<0.01 vs Group Ang II.(n = 5 for each group).

Ang II induced the proliferation of the atrial CFs in both a time and a dose dependent manner. Following 48 h of culture, the proliferation of the Ang II treated cells was greater than that of the control cells. Optical density in different concentration of Ang II 10^−8^, 10^−7^, 10^−6^, 10^−5^, 10^−4^ mol/L was 0.56,0.54,0.59,0.56,0.69 respectively vs 0.06 in control group,(*P*<0.01) (Fig 4A).

The Ang II receptor antagonist Losartan and the RhoA/ROCK-1 inhibitors (Sim and Y27632) significantly inhibited the Ang II induced proliferation of the atrial CFs in a dose dependent manner. Optical density in different concentration of Los 10^−8^,10^−7^,10^−6^,10^−5^,10^−4^ mol/L was 0.35,0.33,0.29,0.29,0.29 respectively vs 0.38 in Ang II(10^-6^mol/L) group,(*P*<0.05). Optical density in different concentration of Simvastatin 0.01μM,0.1μM,1μM was 0.29,0.23,0.21 respectively vs 0.34 in Ang II(10^-6^mol/L) group (*P*<0.05). Optical density in different concentration of Y27632 10^−8^,10^−7^,10^−6^,10^−5^,10^-4^mol/L was 0.30,0.29,0.26,0.28,0.13 respectively vs 0.34 in Ang II(10^-6^mol/L) group (*P*<0.05). (Fig 4B–4D). However, none of the inhibitors had an effect on the proliferation of the atrial CFs in the absence of Ang II stimulation (data not shown). The results indicate that the RhoA/ROCK-1 pathway is a potential downstream effector in Ang II induced atrial CFs proliferation.

Ang II stimulation upregulated the expression of TGF-β1, ROCK-1 and CTGF in a dose dependent manner. The expression mRNA of ROCK-1 in different concentration of Ang II(10^−8^, 10^−7^, 10^−6^) was 1.22±0.02,1.35±0.06,1.73±0.07 respectively at 21 hours vs 0.95±0.01,1.36±0.03,1.59±0.08 at 6 hours (*P*<0.01). The expression mRNA of CTGF in different concentration of Ang II(10^−8^, 10^−7^, 10^−6^) was 0.94±0.01,1.10±0.03,1.20±0.02 respectively at 21 hours vs 0.95±0.02,1.08±0.01,1.08±0.01 at 6 hours (*P*<0.01) (Fig 4E–4G). Each of the inhibitors down-regulated the expression of ROCK-1 and CTGF. The expression mRNA of ROCK-1 in different inhibitors (Sim, Y27632, Los, Los+Sim) was 0.89±0.05,1.27±0.03,1.41±0.03,1.23±0.01 respectively vs 1.60±0.08 in Ang II(10^-6^mol/L) group. The expression mRNA of CTGF in different inhibitors (Sim, Y27632, Los, Los+Sim) was 0.87±0.04,0.91±0.02,1.15±0.01,1.23±0.01 respectively vs 1.20±0.02 in Ang II(10^-6^mol/L) group. Simvastatin and Losartan downregulated the expression of TGF-β1, and their combined administration exerted synergistic effects. Y27632 had no effect on Ang II induced TGF-β1 expression. The results indicated that TGF-β1 and RhoA/ROCK-1 are both downstream factors of Ang II in atrial CFs (Fig 4H–4J).

### 3.6 The RhoA/ROCK-1 pathway mediated TGF-β1 induced CTGF expression in the atrial CFs

**Fig 5:**
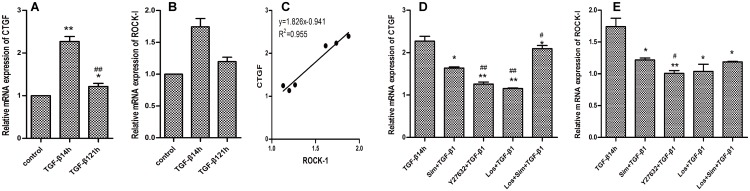
The effects of TGF-β1 on the mRNA expression of CTGF (A) and ROCK-1 (B) in the atrial CFs. (*P<0.05, ** P<0.01 vs control group, #*P*<0.05, ##*P*<0.01 vs TGF-β1 (4 h) group). The correlation between the mRNA expression of ROCK-1 and CTGF (C). The effects of Sim, Y27632, and Los and the combined administration of the inhibitors on the mRNA expression of CTGF (D), ROCK-1 (E) in the atrial CFs stimulated by TGF-β1. *P<0.05,**P<0.01 vs TGF-β1 group, #*P*<0.05, ##*P*<0.01 vs Sim+TGF-β1 group.(n = 5 for each group).

TGF-β1 significantly upregulated the expression of both CTGF and ROCK-1 (Fig 5A and 5B). The expression mRNA of CTGF in different time induced by TGF-β1(4h,21h)was 2.28±0.11,1.22±0.07respectively(vs control group,*P*<0.01). The expression mRNA of ROCK-1 in different time induced by TGF-β1(4h,21h)was 1.74±0.13,1.20±0.07 respectively(vs control group, *P*<0.01). Furthermore, the expression levels of CTGF and ROCK-1 correlated strongly (Fig 5C), which confirmed our hypothesis. Either the single or the combined administration of Sim and Los downregulated the mRNA expression of ROCK-1 and CTGF in the atrial CFs (*P*<0.05), but the combined administration of the inhibitors did not exert synergistic effects. The expression mRNA of ROCK-1 in different inhibitors (Sim,Y27632,Los,Los+Sim)was 1.22±0.03,1.01±0.04,1.04±0.11,1.19±0.01 respectively vs 1.74±0.13 in TGF-β1 group. The expression mRNA of CTGF in different inhibitors (Sim,Y27632,Los,Los+Sim)was 2.27±0.11,1.64±0.03,1.26±0.05,2.09±0.07 respectively vs 2.28±0.11 in TGF-β1 group (Fig 5D and 5E).

### 3.7 Smad2/3 and RhoA were mediators of TGF-β1 in the atrial CFs

**Fig 6:**
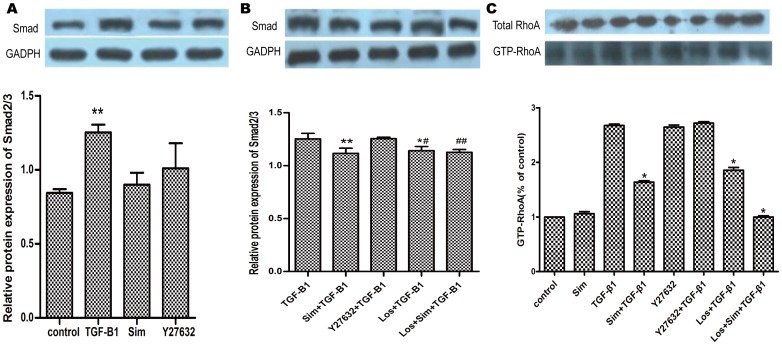
The effect of TGF-β1 on the Smad signal pathway. (A) TGF-β1 upregulated the expression of Smad2/3. (B) Sim and Losinhibited the protein expression of Smad2/3 in the atrial CFs treated with TGF-β1. (C) The ratio of GTP-RhoA/total RhoA following different treatments. *P<0.05, **P<0.01 vs TGF-β1 group, #*P*<0.05, ##*P*<0.01 vs Y27632+TGF-β1 group. (n = 5 for each group).

In the present study, treatment of the atrial CFs with TGF-β1 increased the expression of Smad2/3(1.26±0.05) vs control group (0.83±0.03),(*P*<0.01) (Fig 6A). The expression protein of Smad2/3 in different inhibitors (Sim,Y27632,Los,Los+Sim)was 1.12±0.05,1.26±0.01,1.14±0.04,1.13±0.03 respectively vs 1.25±0.05 in TGF-β1 group. Losartan and Simvastatin attenuated the effects of TGF-β1. Unlike Losartan and Simvastatin, Y27632 had no effect on the expression of Smad2/3 (*p*>0.05) (Fig 6B). Furthermore, treatment of the atrial CFs with TGF-β1 increased the expression of GTP-RhoA. The expression protein of GTP-RhoA in different inhibitors (Sim,Y27632,Los,Los+Sim)was 1.64,2.72,1.86,0.99 respectively. Losartan and Simvastatin attenuated the effects of TGF-β1,inhibited RhoA activity as opposed to RhoA protein expression. Y27632 had no effect on either the expression or the activity of RhoA (Fig 6C).

## 4. Discussion

In this study, we observed that electrical parameters such as AVN-Wen, CSNRT and ERPAV were significantly increased, whereas ERPA was markedly decreased, following 4 weeks of RAP. Meanwhile, the expression of hydroxyproline was increased in the left atrium, and both the left atrial upper-bottom diameter and left atrial left-right diameter were significantly increased. AF is a progressive disease that develops secondary to continuous structural remodeling and electrical remodeling of the atria, often due to AF itself. The development of regional fibrosis results in the electrical uncoupling of adjacent muscle bundles, subsequently resulting in discontinuities in transverse conduction, localized regions of conduction slowing and block, and increased heterogeneity, which ultimately promotes both focal and macro-re-entry[24] and results in increased vulnerability to both AF and LA size changes. LA dilatation has been identified as an independent risk factor for both the development of AF and the aggravation of atrial contractile dysfunction. Patients with persistent AF often experience left atrial enlargement[25].

Desmoplastic material (TGF-β1, CTGF, and Collagen-I) plays an important role in atrial fibrosis. Increased levels of CTGF, Collagen-I and TGF-β1 mRNA and protein expression were observed in the pacing group, findings consistent with those of previous studies. ROCK-1 and RhoA mRNA expression and ROCK-1 protein expression were also increased, which paralleled the increases in desmoplastic material expression. However, whether RhoA and ROCK-1 participate in atrial fibrosis has not been determined. The RhoA/ROCK-1 pathway plays a significant role in myocardial fibrosis[26]. Fluvastatin decreases cardiac fibrosis in spontaneously hypertensive rats[7]. Simvastatin reduces human atrial myofibroblast proliferation independently of cholesterol via the inhibition of RhoA[27]. We observed that ROCK-1 and RhoA were activated in an AF model and were involved in atrial fibrosis. Our data convincingly demonstrated that dogs with AF exhibit increased levels of desmoplastic material compared with dogs in sinus rhythm; these changes correlate with both RhoA and ROCK-1 activity.

CTGF is a downstream factor of TGF-β1[12, 13] and Ang II[12]-induced cardiac fibrosis, and widely regarded as an important amplifier of the profibrogenic action of TGF-β1 in a variety of tissues. Although we observed that RhoA and Rho kinase participate in atrial fibrosis, whether the RhoA/ROCK-1 signaling pathway participates in atrial fibrosis via Ang II and TGF-β1-mediated CTGF upregulation warrants further study. RhoA is a member of the Ras superfamily, the most studied downstream effector of Rho. Rho-kinase mediates signals from Rho and induces the formation of both stress fibers and focal adhesions[28]. Yang et al observed that Ang II-induced cardiac hypertrophy and fibrosis in vivo are at least partially mediated by RhoA, p-38 MAPK and TGF-β1[29]. An FPPS inhibitor attenuated Ang II-induced RhoA activation, p-38 MAPK phosphorylation and TGF-β1 mRNA expression[29]. Deletion or inhibition of ROCK-1 also leads to decreased angiotensin II–induced CTGF expression and cardiac fibrosis[20, 30]. Perivascular fibrosis in hearts was increased to a lesser extent in ROCK1(+/-) mice and associated with decreased expression of CTGF, and type III collagen[20]. Y-27632 is widely used as a specific inhibitor of the Rho-associated coiled-coil forming protein serine/threonine kinase (ROCK) family of protein kinases, which can attenuate cardiac fibrosis in the setting of Ang II-induced cardiac hypertrophy and perivascular fibrosis[31]. CTGF is a downstream factor of RhoA/ROCK in both renal[32] and lung fibroblasts[33], which are regulated by TGF-β1 and Ang II. Furthermore, type I collagen, CTGF and α-SMA are each upregulated via the activation of RhoA signaling in keloid fibroblasts, which may be reversed by Simvastatin[34]. Rho kinase blockade decreased CTGF levels, probably through nuclear factor kappaB inhibition, and caused decreased expression of the type I collagen gene[35]. In the present study, we demonstrated that Ang II not only induced the proliferation of neonatal Sprague Dawley (SD) rat atrial cells but also increased the expression of both ROCK-1 and CTGF in both a concentration and a time-dependent manner. The expression of CTGF correlated strongly with that of ROCK-1. Simvastatin, Y27632 and Losartan modulated the Ang II-induced proliferation of CFs and the expression of CTGF. These findings suggested that CTGF may be a therapeutic target of RhoA/ROCK-1-mediated fibrosis in the development of AF. The RhoA/ROCK-1 signaling pathway participated in atrial remodeling downstream of RAS. The inhibition of RhoA/ROCK-1 signaling via Simvastatin and Y27632 attenuated extracellular matrix deposition by inhibiting both atrial CF proliferation and CTGF mRNA expression. The mechanism underlying the Ang II-mediated CTGF expression involves the activation of the RhoA/ROCK-1 pathway. The inhibition of this pathway blocks CTGF expression.

TGF-β1 mediates atrial fibrosis, and the Smads are important downstream effectors of this process [17]. In 2011, We have demonstrated When Smad2 and Smad3 genes were silenced by siRNA, the Ang II–induced increase in collagen I expression in the fibroblasts was significantly less than that in the fibroblasts transfected with control siRNA[17]. Recent studies have determined that non-Smad pathways such as the RhoA/ROCK signaling pathway are also involved in myocardial fibrosis[20, 26]. TGF-β1-mediated activation of CTGF gene expression is controlled by Smads, and Rho/ROCK signaling in intestinal fibrosis[36]. Specific targeting of RhoA with C3 exotoxin or of the Rho kinases with the inhibitor Y-27632 similarly prevented induction of CTGF after stimulation with TGF-β1[32]. CTGF activates TGF-β1 signals by direct binding in the extracellular space[37]. Blockade of PKCδ by the selective inhibitor Rottlerin or by siRNA knockdown significantly reduced TGF-β1-induced CTGF production[38]. Although similar levels of active TGF-β1 were present in the transgenic atria and ventricles, overt fibrosis was observed only in the atria[10]. Furthermore, transgene-positive mice expressing high levels of either wild-type or activated RhoA exhibited pronounced atrial enlargement, enhanced AF susceptibility and atrioventricular block prior to the onset of ventricular failure[39]. Nevertheless, the relationship between TGF-β1 and the RhoA/ROCK-1 signaling pathway with respect to atrial fibrosis is controversial. We have confirmed that CTGF is a profibrotic molecule induced by Ang II and TGF-β1 via the RhoA/ROCK pathway in CFs. We also investigated the crosstalk between the TGF-β1/Smad and the RhoA/ROCK-1 signaling pathways with respect to the development of atrial fibrosis. Ang II promotes atrial CF TGF-β1 mRNA expression over a period of 6 hours in a concentration-dependent manner, a process characterized by the increased expression of Smad2/3, Collagen-I and CTGF. These results indicated that TGF-β1 may function as an early fibrogenic factor involved in Ang II-mediated fibrosis and that TGF-β1/Smad signaling downstream of Ang II plays an important role in atrial remodeling. Smad proteins are classic signaling cascade receptors of TGF-β1; the blockade of AT1 signaling may downregulate Smad expression[17]. Pre-incubation with Simvastatin decreases the TGF-β1 mRNA expression and RhoA activity induced by Ang II. Compared with the Simvastatin group, TGF-β1 mRNA expression was more significantly decreased in the Losartan group. Both simvastatin and Y27632 significantly downregulated TGF-β1-induced CTGF expression but exerted no significant effects on the cells in the absence of TGF-β1 stimulation. Compared with Simvastatin, Y27632 was a more efficient inhibitor of CTGF expression. Y27632 exerted no significant effect on Smad. Simvastatin attenuated CTGF expression via the inhibition of RhoA activity and TGF-β1 expression. Ang II partially mediated TGF-β1 expression via RhoA activation. ROCK1 contributed to the development of cardiac fibrosis[30]. On the molecular level, our results indicated that two pathways, the Ang II/TGF-β1/Smad pathway and the Ang II/TGF-β1/RhoA/ROCK1 pathway, were associated with atrial remodeling. CTGF was a factor in both TGF-β1/RhoA/ROCK1 and TGF-β1/Smad2/3 signaling.

Ang II activates multiple intracellular signaling molecules and pathways, including the RhoA/ROCK-1 pathway. We have demonstrated that AT1 receptor antagonist inhibited the Ang II-induced increase in TGF-β1 expression in isolated adult rabbit cardiac fibroblasts, and downregulated excessive atrial collagen synthesis in animal left atria after pacing[17]. RhoA/ROCK signaling activated by Ang II in cardiomyocytes and vascular smooth muscle cells and is closely tied to both cardiac myocyte hypertrophy and smooth muscle cell proliferation. The Rho-ROCK signaling pathway may represent a new therapeutic target in the prevention of atrial fibrosis. Our observations indicate that the RhoA/ROCK-1 pathway participates in atrial fibrosis. Simvastatin and Y-27632 attenuate CF proliferation and the expression of both collagen I and CTGF. Blocking RhoA/ROCK-1 signaling may attenuate atrial remodeling. Meanwhile, an ARB and a Rho kinase inhibitor exerted similar effects with respect to the inhibition of collagen-I deposition and CTGF expression in the atrial CFs. Both atrial CF proliferation and CTGF expression were significantly suppressed by the administration of an ARB and a Rho kinase inhibitor. However, the combined effects of these two drugs on atrial fibrosis have not been studied. The present study demonstrated that Simvastatin inhibited Ang II-induced CF proliferation, both alone and in combination with Losartan, and also downregulated the expression of CTGF, hydroxyproline and Collagen-I. TGF-β1, ROCK-1 and CTGF expression, as well as RhoA activity, decreased more significantly in the combination therapy group. The co-incubation of Simvastatin and Losartan exerted synergistic effects. The combination of Simvastatin and Losartan may be a promising therapeutic modality in the attenuation of atrial remodeling.

In conclusion, Ang II/TGF-β1/RhoA/ROCK-1 signaling plays an important role in atrial remodeling. CTGF is an important mediator of the above signaling pathway. RhoA is an additional downstream effector of TGF-β1 and affects the activity of Smad 2/3 and the RhoA/ROCK-I pathway. These findings may provide clinicians and researchers with new insight regarding the mechanisms underlying AF induced atrial fibrosis, as well as information regarding potential therapeutic targets in the management of AF.
